# Impact of Rye Malt with Various Diastatic Activity on Wholegrain Rye Flour Rheology and Sugar Formation in Scalding and Fermentation Processes

**DOI:** 10.3390/foods13132077

**Published:** 2024-07-01

**Authors:** Ruta Murniece, Sanita Reidzane, Vitalijs Radenkovs, Roberts Matisons, Ilona Dabina-Bicka, Dace Klava, Ruta Galoburda

**Affiliations:** 1 Food Institute, Latvia University of Life Sciences and Technologies, Riga Street 22, LV-3004 Jelgava, Latvia; 2 Institute of Horticulture (LatHort), Graudu Street 1, LV-3701 Dobele, Latvia; 3 Division of Smart Technologies, Research Laboratory of Biotechnology, Latvia University of Life Sciences and Technologies, Rigas Street 22b, LV-3004 Jelgava, Latvia; 4 Latvian State Forest Research Institute ‘Silava’, 111 Rigas Str., LV-2169 Salaspils, Latvia

**Keywords:** rye malt, rye flour, rheology, scald, fermentation

## Abstract

Amylase activity in rye flour plays a crucial role in the production of rye bread. When preparing a scald in rye bread production, diastatic rye malt is utilized to augment the amylase activity of the rye flour. This study investigated the effects of the diastatic power (DP) and concentration of rye malt on the Falling Number (FN) and the rheological properties of rye flour. Additionally, it examined reducing sugars in the scalding process and fermentation. Mixolab results provided comprehensive data on dough properties at different temperature stages, highlighting significant changes in starch gelatinization and enzyme activity due to varying malt diastatic power and concentrations. The decline in the gelatinization index (C3-C2) indicated faster starch gelatinization with increased diastatic power. Adding rye malt significantly increased maltose content in the saccharified and fermented scald, promoting a favorable environment for lactic acid bacteria and yeasts. FN and Amylograph results showed that less active malt (DP 170, 179 °WK), at a 1.5% concentration, could achieve similar effects as the more active malt (DP 362, 408 °WK) at 0.5%. Adding rye malt to rye flour allows for the regulation of the flour’s rheological properties and FN, adjustable based on malt DP and concentration.

## 1. Introduction

Rye (*Secale cereale* L.) is spread throughout, and often grown in, northern and eastern Europe. Bread made from rye flour is a traditional bread in the Baltic region [1]. Endogenous amylolytic enzyme activity and starch quality are important indicators in rye flour baking [2]. α-amylase and β-amylase play a crucial role in starch degradation during scald preparation and the fermentation of rye dough. During scald preparation, diastatic rye malt may be added to rye flour and mixed with hot water to promote enzymatic activity [3]. The active enzymes efficiently break down starch into fermentable sugars, serving as nutrients for lactic acid bacteria (LAB) and yeasts [4,5]. The ability of diastatic malt enzymes to convert starch into sugars influences gas production, water absorption, dough rheology, and handling properties, impacting the final bread texture [6,7].

The diastatic activity of malt, which reflects the combined action of α-amylase, β-amylase, and limit dextrinase, is critical for further hydrolyzing starch and damaged starch into fermentable sugars [8]. The activity is expressed as a diastatic power, and it indicates the concentration of total enzymes that break down starch and is an important indicator of malt quality [9]. The malting conditions can significantly affect the increase in diastatic activity in the malt [10]. The recommended amount of malt additive ranges from 0.2% to 2% [11,12], depending on its diastatic power, the quantity added, and the flour quality.

Both α-amylase and β-amylase play an important role in starch degradation. α-Amylase randomly hydrolyzes the α-(1,4)-linkages within the starch chain, producing dextrins, maltose, and glucose, resulting in a rapid decrease in the viscosity of starch solution [13]. Dextrins serve as substrates for β-amylase, which releases maltose from the non-reducing ends of the starch chain, promoting saccharification. β-Amylase is considered to be the important enzyme that affects diastatic power and provides fermentable sugars [14]. The sugar content in flour and dough plays a very important role in determining the rate and intensity of fermentation, with glucose and fructose being the most used sugars, followed by maltose and sucrose [15]. In rye grains, sugars constitute about 3% of the total carbohydrates, with sucrose making up 2.58%, glucose 0.61% and fructose 0.18% [16]. Given the relatively small natural sugar content in grains and flour, most of the sugar required for fermentation is produced during the enzymatic degradation of damaged starch [17]. The optimum temperature for α-amylase activity is 72–74 °C [18], while for β-amylase it is 60–65 °C, both of which correspond to typical scald conditions. The scalding process enhances this saccharification by creating optimal conditions for amylase activity, causing starch to swell and allowing easier enzyme access. The elevated temperatures during scalding ensure that amylases work efficiently, maximizing the breakdown of starch into sugars [4].

The Falling Number (FN) test assesses the amylase activity influencing starch degradation; however, starch properties, including granule size and structure, also affect FN outcomes, making enzyme activity and starch characteristics pertinent to the test [19,20]. For baking high-quality rye bread, the FN range is considered to be between 125–200 s [21]. α-Amylase activity formed during ripening and harvest time determines the variation in FN, which usually ranges from 118 to 436 s [22,23]. Rye four with low amylase activity results in a more viscous flour–water mixture, indicating a reduced ability to ferment and form fermentable sugars, and corresponds to a high FN (above 200 s) [21]. Conversely, flour with a low FN (below 100 s) indicates high enzymatic activity, which enhances the starch degradation–hydrolysis into fermentable sugars and dextrins, but also leads to a dough that is difficult to process and a sticky bread crumb [24,25]. Although the Falling Number method is widely used by bakers, grain growers, and millers, it does not directly reflect the enzymatic activity of flour. Instead, it describes the properties of starch, which are of great importance for rye bread production [26].

The starch, comprising 75–80% of rye flour, serves as the predominant component that influences both the technological properties of the dough and the texture of the bread crumb. The primary starch characteristics that can be determined instrumentally, e.g., amylographically, include the maximum viscosity, the temperature at the maximum viscosity, and the starch gelatinization temperature [26]. The maximum viscosity is related to enzyme activity and largely correlates with the FN. The α-amylase activity affects the liquefaction dynamics of the starch paste during heating, with higher α-amylase activity resulting in lower maximum viscosity on the amylograph curve [27]. Additionally, the amount of damaged starch significantly affects the pasting properties and is heavily influenced by milling conditions [17].

The effect of rye malt diastatic activity and the concentration of added rye malt on the properties of rye flour provided opportunities to stabilize and maintain the technological processes of scalding and fermentation in rye bread production. Additionally, determining the sugar profile allowed for selecting the optimal amount of malt for the scald saccharification and fermentation processes. Therefore, this study aimed to evaluate the effect of rye malt diastatic power on the Falling Number and rheological properties (maximum viscosity and dough properties using Mixolab (CHOPIN Technologies, Villeneuve la Garenne, France)) of wholegrain rye flour, as well as on sugar formation during the saccharification and fermentation processes in scalding and scald fermentation. Additionally, the enzymatic activities of α-amylase and β-amylase in different malt samples and flour were determined.

## 2. Materials and Methods

### 2.1. Materials

Wholegrain rye flour (further referred to as rye flour in the text), with varying enzyme activities, was tested in this study. The characteristic parameters of rye flour, provided by the manufacturer, are listed in Table 1. The flour was produced from rye grains (variety Kaupo) harvested in 2021 from the Kelmeni farm in Ranka, Latvia. These grains were milled in stone mills at the Kanepites farm in Koceni, Latvia in 2022.

Rye malt flour (further in text, rye malt) with varying diastatic activities (DP ranging from 170 to 408 (°WK)) was used. Samples of rye malt were obtained from LPKS LATMALT (Stalgene, Latvia) and they were stored in sealed containers in the dark at room temperature for subsequent analysis. The codes and characteristics of malt samples are provided in Table 2.

Rye malt samples at three concentrations (0.5, 1.0 and 1.5%) were mixed with each rye flour sample, resulting in a total of 45 flour–malt blends. The samples were mixed for 15 min using a Teddy mixer (A/S Varimixer, Brøndby, Denmark) and then stored in the sealed containers in the dark at room temperature for subsequent analysis.

### 2.2. Determination of α-Amylase and β-Amylase Activity of Rye Flour, Malt and Flour–Malt Samples

Megazyme kits (Megazyme Ltd., Wicklow, Ireland) were used to determine the enzymatic activity of both flour (Table 1) and malt (Table 2). The α-amylase activity (expressed in Ceralpha Units, CU g^−1^) was determined using the Ceralpha method according to AACC 22.-02.01, while the β-amylase activity (expressed in Betamyl-3^®^ units, BU g^−1^) was determined using the BETAMYL-3^®^ method. Enzyme extraction and activity measurements were performed following the manufacturer’s instructions in triplicates, utilizing BioTek Synergy H1 Multimode Reader (Agilent Technologies, Winooski, VT, USA) at 400 nm.

### 2.3. Falling Number Determination of Rye Flour and Flour–Malt Samples

The FN was determined for both flour and flour–malt blend samples following the AACC 56-81.03 standard [28]. All tests were made in triplicates.

### 2.4. Analysis of Rye Flour, Malt and Flour–Malt Rheological Properties Using an Amylograph

The rheological properties of rye flour and rye flour–malt blends were evaluated using the Brabender Viscograph-E (Brabender GmbH & Co, Duisburg, Germany). The maximum viscosity (in BU) of the rye flour–malt blends was measured according to ICC standard method No. 126/1 [29]. All tests were made in duplicate.

### 2.5. Analysis of Rye Flour, Malt and Flour–Malt Rheological Properties Using Mixolab

The rheological properties of both rye flours and rye flour–malt blends were evaluated using the Mixolab 2 (CHOPIN Technologies, Villeneuve la Garenne, France) according to the Chopin+ protocol (ICC 173-1) [30]. 

The parameters were obtained, including water absorption, maximum consistency at 30 °C (C1), dough weakening (C2), time to reach dough weakening (TC2), peak torque (C3), stability of hot dough at 90 °C (C4), and final torque (C5).

### 2.6. Scald Preparation and Fermentation

To evaluate the effect of malt in the technological process, rye wholegrain flour scald was prepared following the previously outlined procedure [31], with slight modifications. The stages and sequence of operations are outlined below.

1. Scalding and Saccharification: RF2 flour and malt with various DP (DP—170, 261, 408 °WK) at concentrations of 0.5%, 1.0%, 1.5% were used (Table 3). A mixture of rye flour and malt was scalded with water at 96 °C and mixed for 15 min at 300 rpm (speed 2) using a Teddy mixer (A/S Varimixer, Brøndby, Denmark). Post-mixing, the dough equilibrates to a temperature of 75 °C. Controlled cooling follows, with a gradual decrease in the temperature from 75 °C to 55 °C in the climate chamber Memmert ICH 110 (Memmert GmbH, Schwabach, Germany). This thermal regimen was optimized for the activity of α-amylase and β-amylase, ensuring the development of a fermentable substrate for the fermentation stage. The scald sample was taken for lyophilization.

2. Fermentation: At a maintained temperature of 55 °C, 22 g of fermented scald (obtained from bakery “Kelmeni”), containing *Lactobacillus delbrueckii* as indicated by the manufacturer, was introduced to the scald after saccharification. The temperature was progressively reduced to 28 °C, over 20 h. This step is crucial for the metabolic activities associated with the fermentation process. Upon completion of fermentation, a sample of the fermented scald was lyophilized for further analysis.

### 2.7. Determination of pH and TTA in Fermented Scald

The pH of the fermented scald was measured using a pH meter (Mettler Toledo, Germany) following the standard method AACC 02-52:1999 [32]. Total titratable acidity (TTA) was analyzed following the AACC 02-31.01 standard [33].

### 2.8. Sample Preparation for Sugar Profile Analysis

Scald samples were lyophilized in the freeze–dryer FT33 (Armfield Ltd., Hampshire, UK) with a condenser camber set at −40 °C and 6.4 Pa for 72 h. Following freeze–drying, the samples were ground using a Foss Knifetec 295 Mill laboratory mill (Foss Analytical Co., Ltd., Suzhou, China).

### 2.9. Sugar Profile Evaluation in Scald after Saccharification and Fermentation 

#### 2.9.1. Sample Preparation for Carbohydrate Analysis

The process of isolating mono- and disaccharides from the grain-derived products involved gently heating the sample at 60.0 °C for 30 min., followed by ultrasound treatment at a frequency of 50 kHz and an output power of 360 W for 30 min., and at a temperature of 25.0 ± 1 ° C using an Ultrasons ultrasonic bath (J.P. Selecta^®^, Barcelona, Spain) according to the methodology described by Radenkovs et al. [34]. Briefly, a sample (1.0 g ± 0.1) was introduced into a 15.0 mL conical centrifuge tube (Sarstedt AG & Co. KG, Numbrecht, Germany), and 10.0 mL of 50% acetonitrile (H_2_O:CH_3_CN, *v*/*v*) was then added. Subsequently, the resulting mixture was intensively vortex-mixed with a ZX_3_ vortex mixer (Velp^®^ Scientifica, Usmate Velate, Italy). Once completed, the prepared samples were centrifuged at 4500 rpm for 10 min. (3169× *g*) at a temperature of 19.0 ± 1 °C using a Sigma centrifuge, 2-16KC (Osterode near Harz, Germany) to separate the fractions. Before high-performance liquid chromatography–refractive index detection analysis (HPLC-RID), the collected supernatant was filtered through a hydrophilized polytetrafluoroethylene membrane filter (CROMAFIL^®^ Xtra H-PTFE) with a pore size of 0.45 μm (Macherey-Nagel GmbH & Co. KG, Düren, Germany).

#### 2.9.2. Analytical Conditions of HPLC-RID for Quantitative Analysis of Carbohydrates

Quantitative analysis of mono- and disaccharides in the prepared samples was conducted using a Waters Alliance HPLC system (model No. e2695) with a 2414 Refractive Index detector and a 2998 column heater (Waters Corporation, Milford, MA, USA). Chromatographic separation employed an Altima Amino (4.6 × 250 mm; 5 μm; Grace™, Columbia, MD, USA) column with a maintained column and flow cell temperature of 30 °C. The mobile phase, a mixture of double distilled water and acetonitrile (80:20, *v*/*v*), operated in an isocratic mode at a flow rate of 1.0 mL min^−1^ with an injection volume of 15 μL. System control, data acquisition, analysis, and processing were all handled using Empower Chromatography Data Software version Empower 3 Build 3471 (Waters Corporation, Milford, MA, USA).

### 2.10. Statistical Analysis

Given the orthogonal experimental design and sample size, simple linear models were used to evaluate the effects of malt activity and malt concentration, as well as their interaction on FN according to the experimental setup. Considering that only a few levels of malt activity and concentration were analyzed, these variables were treated as factorial.

The model in general form was as follows:

yi j = µ + Fi + Cj + Fi:Cj + ε,(1)

where Fi is the fixed effect of malt activity (factorial non-ordered, three levels), Cj is the fixed effect of concentration of rye malt (factorial non-ordered, three levels), Fi:Cj is the interaction of the fixed effects to evaluate the consistency of individual contributions, and the linearity and complexity of the relationships. 

The levels of the significant predictors were compared using the Tukey’s HSD post hoc test. The conformity of the model with the statistical assumptions was evaluated using the diagnostic plots; the normality was also evaluated by the Shapiro–Wilk test. To evaluate the strength of the relationships among α-amylase and β-amylase activities, as well as the results of the Mixolab curve, maximum viscosity, FN, and sugars, Pearson correlation analysis was used. Data analysis was conducted using R software, v. 4.4.0 [35].

## 3. Results and Discussion

### 3.1. Evaluation of Malt Diastatic Activity

**Figure 1:**
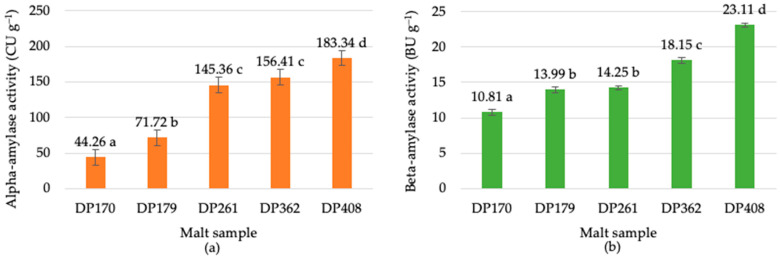
α-Amylase (**a**) and β-amylase (**b**) activity of five rye diastatic malt samples with various diastatic power (DP): 170, 179, 261, 362, 408 (°WK). Bars represent the mean value of three replicates; error bars represent the confidence interval at confidence level of 95%. Different letters (a–d) on bars indicate significant differences (*p* ≤ 0.05) between the samples.

In different malt samples with DP 170–408 °WK, α-amylase activity ranged from 44.26–183.34 CU g^−1^ (Figure 1a), and β-amylase activity from 10.81–23.11 BU g^−1^ (Figure 1b). In rye flour, α-amylase activity was not detected, but β-amylase activity was from 7.13 to 8.39 BU g^−1^. Compared to rye malt, the activity of both amylases in rye flour was very low or undetectable, as also observed by Balcerek et al. [18]. Various factors influence the enzymatic activity in rye diastatic malt during the malting process, which impacts the malt’s overall quality and diastatic power. During the grain germination process, factors such as germination temperature, duration, and humidity play crucial roles in enzyme development [10,14].

A strong correlation was observed between α-amylase activity and malt diastatic power (r = 0.87). No significant difference (*p* > 0.05) in α-amylase activity was found for malts at DP261 and DP362. Similarly, β-amylase activity significantly correlated with malt diastatic power (r = 0.88), while the activities for DP179 and DP261 did not differ significantly (*p* > 0.05). These results suggest that diastatic power significantly influences amylase activities in rye malts, with certain DP ranges showing uniform enzyme activity. 

### 3.2. Effect of Malt Diastatic Power on the Rheological Properties of Rye Flour

#### 3.2.1. Changes in the Falling Number of Rye Flour Depending on the Diastatic Activity and Concentration of Malt

**Figure 2:**
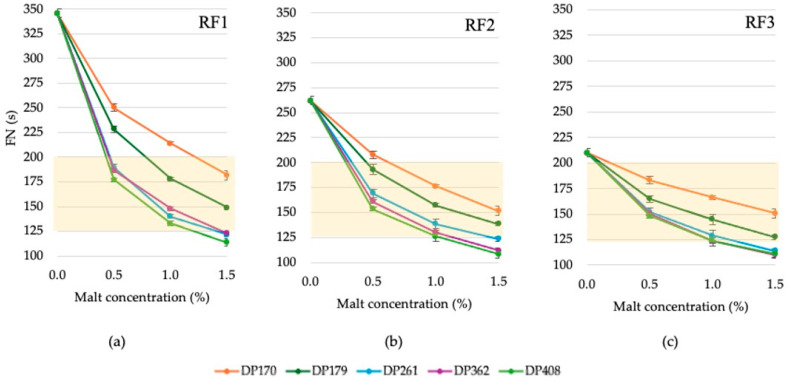
The effect of different concentrations (0.5%, 1.0%, 1.5%) of malt with various diastatic power (DP): 170, 179, 261, 362, 408 (°WK) on rye flour RF1 (**a**), RF2 (**b**), RF3 (**c**) of various Falling Numbers (FN—346, 262, 210 (s), respectively). Error bars represent the confidence interval at a confidence level of 95%. The yellow highlighted background indicates the optimal FN zone.

The effect of varying activities and concentrations of rye diastatic malt on the Falling Number (FN) of rye flour is depicted in Figure 2. The addition of malt with different activities significantly reduced the FN of rye flour, indicating increased enzymatic activity. This trend is consistent across all samples: as malt activity and concentration increased, the FN decreased, reflecting a clear rise in enzymatic activity. Additionally, higher DP malt had a more pronounced positive effect on the flour–malt blend.

In scalding technology for bread preparation, promoting starch hydrolysis through flour’s enzymatic activity is crucial. Combining RF1 flour with malt at DP 170 °WK (0.5% and 1.0%) and DP 179 °WK (0.5%) may not achieve the expected activity levels (Figure 2a,b). Notably, adding malt with lower DP at 1.5% (e.g., RF2: DP170; DP179) can produce a similar effect as higher DP malt (e.g., RF2: DP362; DP408) at 0.5%. This suggests that using a smaller concentration of more active malt can achieve the same enzymatic effect. Detailed linear models (multiple regression) for each rye flour sample (RF1, RF2, RF3) are provided in the appendix to model the FN of rye flour–malt blends at different concentrations using various malt activities (Table S1).

**Figure 3:**
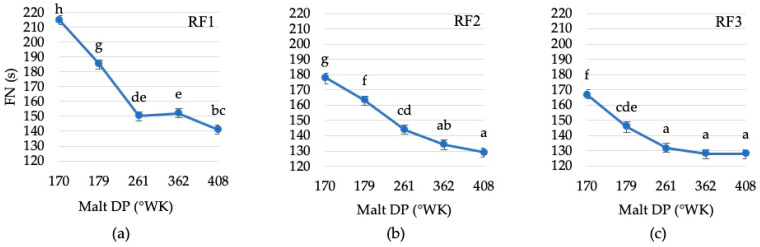
Marginal estimated mean values of malt with different DP effects on the Falling Number of rye flour RF1 (**a**), RF2 (**b**), RF3 (**c**). Error bars represent the confidence interval at a confidence level of 95%. Different letters (a–h) on points indicate significant differences (*p* ≤ 0.05) between the values.

The results showed significant differences in the effect of malt DP on the FN (Figure 3). However, it was observed that there are no significant differences among some high-activity malts between some samples.

The effect of malt on flour sample RF1 showed that malts with DP 261 and 362 °WK did not yield significantly different results (Figure 3a). For flour sample RF2, there was no significant difference between malts with DP 362 and 408 °WK (Figure 3b). Similarly, for flour sample RF3, malts with DP 261, 362, and 408 °WK did not show significant differences (Figure 3c).

#### 3.2.2. Variation in the Maximum Viscosity of Rye Flour Depending on the Diastatic Activity and Concentration of Malt

**Figure 4:**
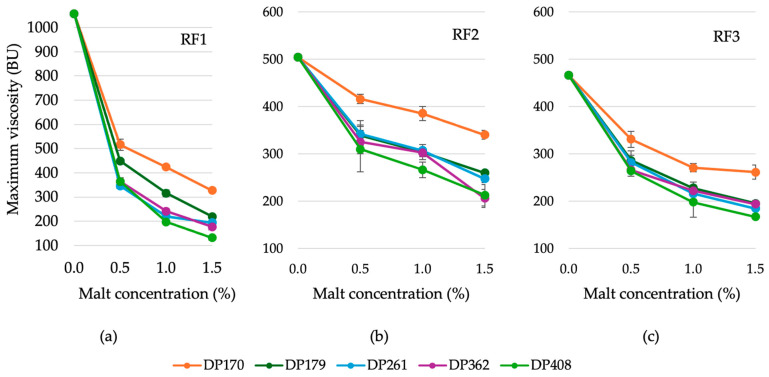
Effect of rye diastatic malt with various diastatic power (DP): 170, 179, 261, 362, 408 (°WK) in different concentrations, % (0.5; 1.0; 1.5) on maximum viscosity of rye flour RF1 (**a**), RF2 (**b**), RF3 (**c**) (FN—346, 262, 210 (s), respectively).

Adding diastatic malt to rye flour significantly influenced the maximum viscosity (Figure 4). Increasing the malt concentration decreased the maximum viscosity, as higher enzymatic activity promotes polysaccharide breakdown, including starch [36,37]. For instance, malt with a lower diastatic power (170 °WK) at 1.5% produced a maximum viscosity similar to that of higher activity malt (408 °WK) at 0.5%. In scalding technology, high viscosity complicates processing and delays fermentation [2]. This similarity highlights the importance of considering both the concentration and enzymatic potency of diastatic malt to achieve specific viscosity in rye flour doughs.

Viscosity is influenced by the properties of the starch–amylolytic complex [22]. Swollen starch granules undergo dissolution due to the melting of amylose, which predominantly occurs during gelatinization, thereby making the soluble fraction accessible to amylases [23]. In addition to starch transformations, there occurs a swelling of pentosans, which facilitates enzymatic hydrolysis by endoxylanases [38]. The xylanase activity in rye flour is assessed to be low [39]. However, Salmenkallio-Marttila et al. [2] have demonstrated that the rye flour xylanases exhibit a more pronounced effect on the rheological properties of flour–water suspension during heating compared to α-amylases. As reported by Peng et al. [36], wheat malt xylanases degrade pentosans into oligosaccharides, significantly reducing the viscosity of water-extractable arabinoxylans. The beneficial effect of pentosans in dough preparation is attributed to their viscosity that they form in the liquid fraction of the dough [40]. 

#### 3.2.3. Characteristics of Flour and Flour–Malt Blend Rheological Properties Using Mixolab 

The results indicate differences in water absorption (WA) between the rye flour samples (RF1, RF2, and RF3 exhibiting WA of 67.0%, 62.0%, and 63.3%, respectively). Adding malt resulted in a slight decrease in water absorption (WA) for all the samples (Table S2). Similar WA results for wholegrain rye flour have been found in other studies, which are in the range of 65–68.5% [41,42,43]. According to Jaksics et al. [41], the rheological properties of flours with similar chemical compositions can differ as a result of enzyme activity.

**Figure 5:**
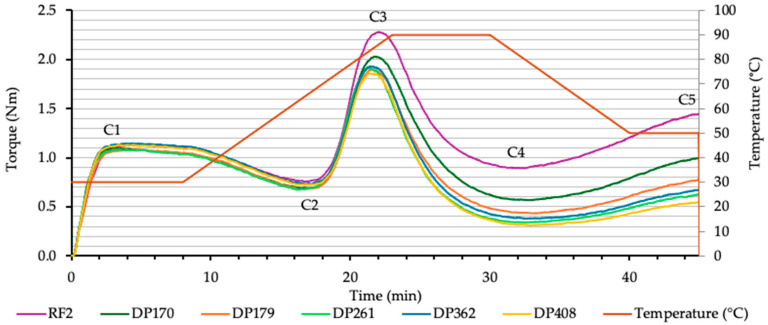
The effect of rye diastatic malt of various diastatic powers (DP): 170, 179, 261, 362, 408 (°WK) and concentration of 1.0% on rye flour RF2 (FN—262 s) Mixolab results (curves). Mixolab profile of dough chracteristics: C1—hydration of dough, C2—dough weakening, C3—starch gelatinization, C4—starch breakdown, C5—starch retrogradation.

The rheological properties of flour–malt blends exhibited significant variability in the starch component during heating and cooling. Analyzing the torque at C3, C4 and C5, significant differences were observed between the rye flour samples (Figure 5). Both malt concentration and diastatic power significantly influenced the starch gelatinization (C3), liquefaction (C4) and retrogradation (C5) (*p* < 0.05). 

**Figure 6:**
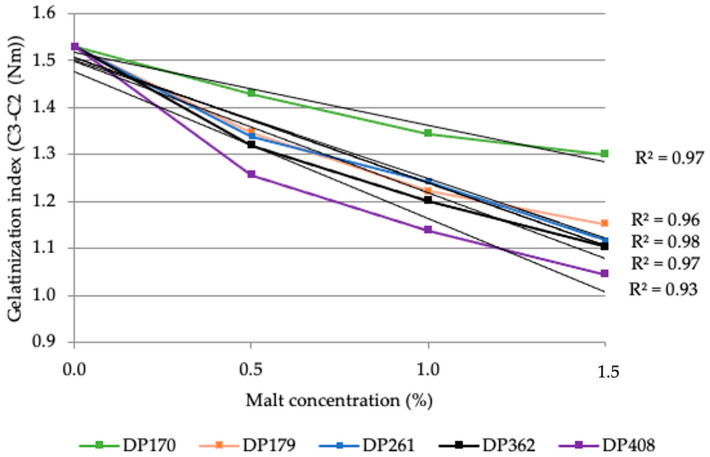
Effect of rye diastatic malt with various diastatic power (DP): 170, 179, 261, 362, 408 (°WK) in different concentrations, % (0.5; 1.0; 1.5) on rye flour RF2 (FN—262 s) gelatinization index (Nm).

The decline in the gelatinization index (C3-C2) indicates a faster starch gelatinization process due to the increased diastatic power of the added malt (Figure 6). The heightened enzymatic activity accelerates gelatinization, as shown by the significant decrease in the C3–C2 index.

This acceleration is likely attributable to the malt enzymes, primarily amylases, rapidly hydrolyzing the starch, thereby reducing the dough’s resistance to gelatinization. The most notable reduction in the gelatinization index was observed in samples with malt diastatic powers of 170 and 179 (°WK). Beyond this range, the reduction was less pronounced, particularly among samples with diastatic powers of 261, 361, and 408 (°WK). The diminished impact on the gelatinization index at higher diastatic powers could indicate a plateau effect, suggesting that enzymatic activity reaches a saturation point. Beyond a certain concentration of enzymes, additional enzymes have a diminished effect on starch breakdown during the gelatinization phase, as measured by the Mixolab.

**Figure 7:**
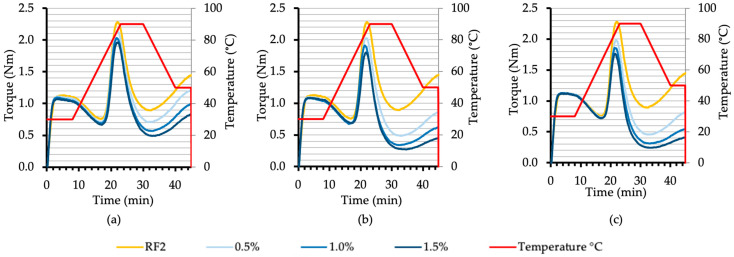
The effect of different concentrations (0.5, 1.0, 1.5%) of rye diastatic malt with various diastatic power (DP): 170 (**a**), 261 (**b**), 408 (**c**) (°WK) on rye flour RF2 (FN—262 s) Mixolab results (curve).

As the concentration of malt increased, the availability of enzymes rose, leading to a more rapid and extensive starch breakdown (Figure 7).

The diastatic power of malt is closely related to gelatinization and retrogradation processes in baking [2]. Gelatinization refers to the swelling and hydration of starch granules when exposed to heat and moisture during baking. Diastatic power, through enzymatic activity, contributes to the breakdown of starches into simpler sugars during gelatinization. Retrogradation, on the other hand, involves the reassociation of gelatinized starch molecules, leading to the staling of the bread. Proper enzymatic activity, influenced by diastatic power, can help control retrogradation and prolong the freshness of the rye bread [44].

### 3.3. The Effect of Malt on Sugar Profile Formation during Saccharification and Fermentation of Scald

#### 3.3.1. Sugar Profile of Malt and Flour

According to the results (Table 4), rye flour contained 0.08 g 100 g^−1^ of fructose. The observed values are consistent with those reported by Pejcz et al. [45], indicating the range of fructose from 0.052 to 0.48 g 100 g^−1^. However, Klupsaite et al. [5]’s findings suggested the absence of fructose in rye flour. The presence of glucose in rye flour was also revealed in this study, corresponding to 0.06 g 100 g^−1^, which is aligned with the value highlighted by Pejcz et al. [45], corresponding to 0.132 g 100 g^−1^. The concentration of maltose in rye flour was found to be 0.05 g 100 g^−1^, contrasting with Klupsaite et al. [5]’s finding, indicating the amount of maltose up to 1.02 g 100 g^−1^.

The sugar composition in malt with different DP ranged from 0.08 to 0.09 g 100 g^−1^ fructose, 0.5–0.75 g 100 g^−1^ glucose, 0.82–1.29 g 100 g^−1^ maltose, and 2.31–3.18 g 100 g^−1^ sucrose. Notably, malt with DP 408 exhibited the highest sucrose content.

A blend of RF2 flour and malt with DP 170, 261, and 408 was prepared at concentrations of 0.5%, 1.0%, and 1.5% to assess the impact of saccharification and fermentation on sugar changes in the scald. This approach aimed to capture the diverse effects of malt. The FN of RF2 flour is typical for rye flour and, when combined with malts of varying activity, yields favorable results for bread baking.

#### 3.3.2. Fructose

**Figure 8:**
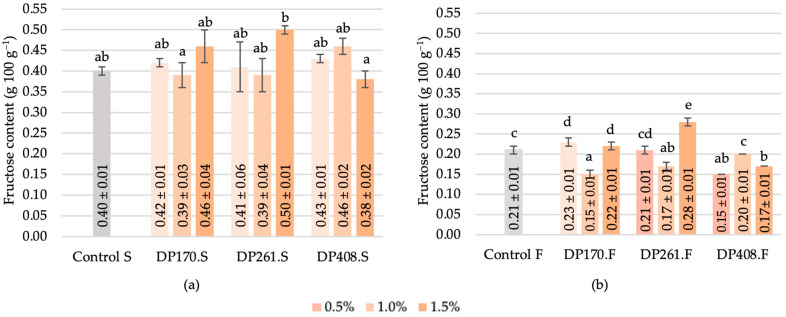
Fructose content in scald after saccharification (**a**) and after fermentation (**b**). Different letters (a–e) on bars indicate significant differences (*p* ≤ 0.05) between the samples. Control S—rye flour RF2 scald without added malt after saccharification; DP170.S, DP261.S, DP408.S—samples of scald prepared from different flour–malt blends after saccharification; Control F—rye flour RF2 scald without added malt after fermentation; DP170.F, DP261.F, DP408.F—samples of scald prepared from different flour–malt blends after fermentation.

The amount of fructose in the samples after saccharification varied from 0.38 ± 0.02 (DP408 1.5%) to 0.50 ± 0.01 (DP261 1.5%), with the control at 0.40 ± 0.01 (Figure 8a). The variations in fructose content during scalding could be attributed to the conversion of fructooligosaccharides into simpler sugars, particularly fructose. Enzymatic activity during scalding was responsible for breaking down complex carbohydrates, including fructooligosaccharides, into monosaccharides like fructose. Based on the findings, there was a decrease in fructose content following fermentation. The most significant decline in fructose content by 29% and 19% was observed in the case of 0.5% and 1.5% of malt addition with DP408 (Figure 8b). This decline can be attributed to the ability of LAB to utilize fructose as a substrate for their metabolic processes. The metabolism of fructose by LAB leads to the production of lactic acid, contributing to the acidification and preservation of the fermented product. These results align with the findings of Pejcz et al. [45], which indicated a reduction in fructans during 48 h spontaneous fermentation and fermentation with *Lactiplantibacillus plantarum* and *Lacticaseibacillus casei*. Interestingly, the addition of 1% malt with DP261 promoted a 19% reduction in fructose during fermentation, while adding 1.5% malt with the same diastatic power contributed to a 33% increase (Figure 8b). These fluctuations may indicate LAB’s preference for using fructose as a carbon source, suggesting their selectivity in utilizing different sugars for nutrition.

#### 3.3.3. Glucose

**Figure 9:**
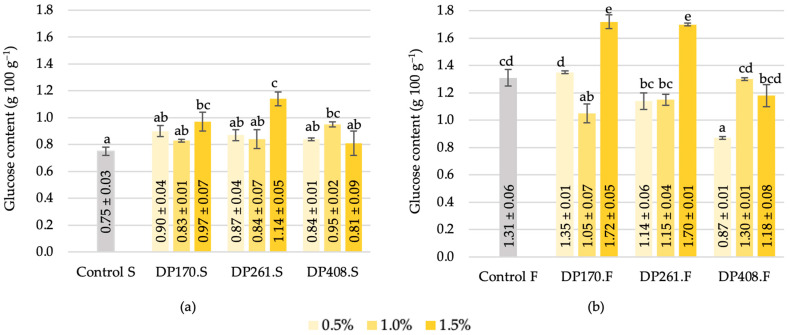
Glucose content of scald after saccharification (**a**) and after fermentation (**b**). Different letters (a–c in (**a**) and a–e in (**b**)) on bars indicate significant differences (*p* ≤ 0.05) between the samples. Control S—rye flour RF2 scald without added malt after saccharification; DP170.S, DP261.S, DP408.S—samples of scald prepared from different flour–malt blends after saccharification; Control F—rye flour RF2 scald without added malt after fermentation; DP170.F, DP261.F, DP408.F—samples of scald prepared from different flour–malt blends after fermentation.

During the saccharification process, glucose content significantly increased, mirroring the trend in fructose levels (Figure 9a). Adding 1.5% malt with DP261 resulted in the highest elevation, with a 52% increase compared to the control sample (Figure 9a). This increase can be attributed to the gradual hydrolysis of starch, leading to the formation of dextrins, glucose, and maltose catalyzed by amylases [46]. The continuous activity of α-amylase facilitates the breakdown of these compounds. Additionally, Klupsaite et al. [5] noted a 13.2% increase in glucose concentration in the scald after 24 h of fermentation. It was observed that fermentation, similar to saccharification, contributed to the rise in glucose content (Figure 9b). The most substantial increase in glucose content was observed when 1.5% of malt with DP170 and DP261 was added to the formulation. The observed changes in glucose content underscore the impact of the specific activity of malt on the saccharification and fermentation processes.

#### 3.3.4. Sucrose

**Figure 10:**
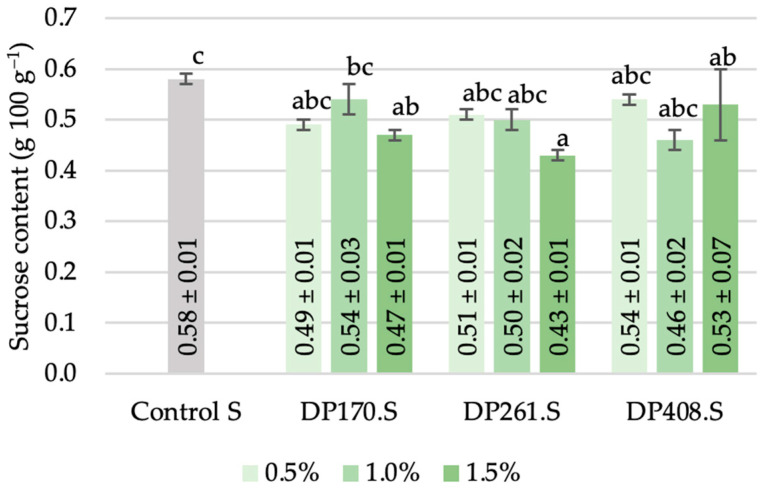
Sucrose content of scald after saccharification. Different letters (a–c) on bars indicate significant differences (*p* ≤ 0.05) between the samples. Control S—rye flour RF2 scald without added malt after saccharification; DP170.S, DP261.S, DP408.S—samples of scald prepared from different flour–malt blends after saccharification.

The sucrose concentration in rye flour–malt blends varied from 0.43 to 0.57 g per 100 g, with the sample without malt exhibiting the highest value, and the blends containing 1.5% and 1% malt with DP261 and DP408 after saccharification, respectively, showing the lowest content (Figure 10). However, no sucrose was detected in any of the sourdough samples after fermentation, indicating the preference of heterofermentative LAB to utilize sucrose as a carbon source during the initial stage of fermentation [47]. A similar observation was noted by Klupsaite et al. [5], who reported the absence of sucrose after 24 h of rye wholemeal flour fermentation with *Lactiplantibacillus paracasei*.

#### 3.3.5. Maltose 

**Figure 11:**
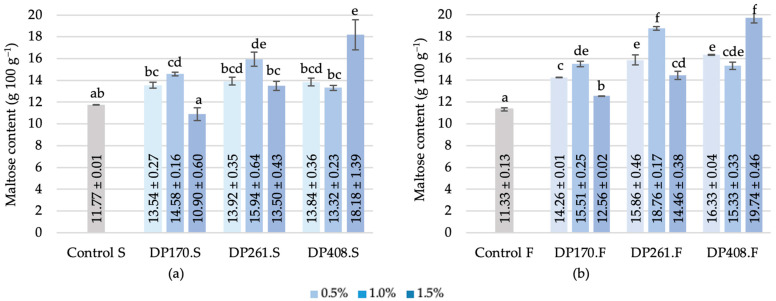
Maltose content of scald after saccharification (**a**) and after fermentation (**b**). Different letters (a–e in (**a**) and a–f in (**b**)) on the bars indicate significant differences (*p* ≤ 0.05) between the samples. Control S—rye flour RF2 scald without added malt after saccharification; DP170.S, DP261.S, DP408.S—samples of scald prepared from different flour–malt blends after saccharification; Control F—rye flour RF2 scald without added malt after fermentation; DP170.F, DP261.F, DP408.F—samples of scald prepared from different flour–malt blends after fermentation.

The concentration of maltose in rye flour and malt blends ranged from 11.8 to 18.2 g per 100 g, with the sample lacking malt exhibiting the lowest value and the blend containing malt with DP408 after saccharification demonstrating the highest concentration (Figure 11a). The most significant increase in maltose content occurred when malt with the activity of DP261 and DP408 was added to the formulation, indicating ongoing starch cleavage and α- and β-amylase activity during saccharification. 

The optimal and controlled conditions during saccharification, particularly temperature, positively contributed to the increase in glucose and maltose released by α- and β-amylase action. Maltose formed during saccharification creates a favorable environment for LAB, contributing to the desired properties of the bread. Previous studies have also observed an increase and dominance of the maltose fraction in scalded rye flour [5,44]. Similar to saccharification, the fermentation of rye flour–malt blends positively contributed to maltose formation (Figure 11b). The most significant increase in maltose content was observed after fermenting blends containing 1% and 1.5% malt with the activity of DP261 and D408, respectively, confirming the excellent properties and optimal dosage of malt required to achieve the desired maltose levels for LAB during fermentation.

#### 3.3.6. Total Sugars

**Figure 12:**
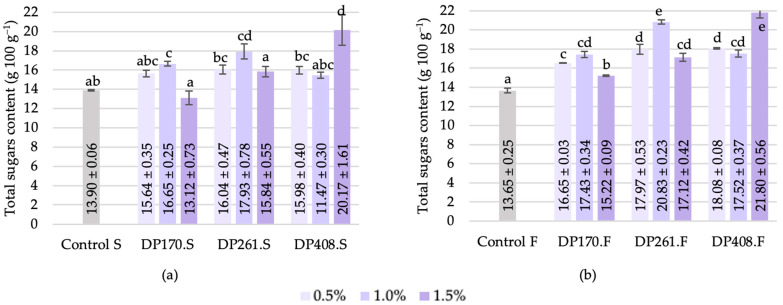
Total sugar content of scald after saccharification (**a**) and after fermentation (**b**). Different letters (a–d in (**a**) and a–e in (**b**)) on bars indicate significant differences (*p* ≤ 0.05) between the samples. DP170.S, DP261.S, DP408.S—samples of scald prepared from different flour–malt blends after saccharification; DP170.F, DP261.F, DP408.F—samples of scald prepared from different flour–malt blends after fermentation.

Following the saccharification stage, α-amylase activity showed a statistically significant correlation (*p* < 0.05) with maltose (r = 0.56) and total sugar content (r = 0.57) (see Figure 12). It is important to note that both maltose and total sugar had an R^2^ close to 1, indicating that the correlation of α-amylase activity with total sugar was primarily influenced by maltose. Notably, β-amylase activity demonstrated a similar pattern to α-amylase activity, with maltose (r = 0.54) and total sugars (r = 0.54) being the only variables that correlated. 

The scald fermentation process resulted in the attainment of parameters characteristic of the spontaneous sourdough. The total titratable acidity (TTA) of scald after fermentation fell within the range of 18.9 to 20.4 mL, which was notably higher compared to the control sample that did not contain added malt (18.3 mL). The pH values ranged from 3.65 to 3.75, indicating that the addition of malt had no significant impact on the pH levels. However, the presence of available sugars in the fermented scald samples implied the potential for ongoing fermentation.

## 4. Conclusions

The interplay between amylase activity and starch characteristics in rye flour is crucial for producing rye bread, particularly with the scalding process. Incorporating diastatic rye malt into the rye flour can effectively modulate its amylase activity. Achieving a specific Falling Number in the flour–malt blend requires the careful consideration of both the diastatic power and concentration of the malt. Depending on the Falling Number of the rye flour, adding malt can decrease it based on its diastatic power and concentration. Malt with higher diastatic power values of 362 and 408 (°WK) at a 0.5% concentration has a similar effect to malt with diastatic power values of 170 and 179 (°WK) at a 1.5% concentration. Consequently, incorporating malt reduces maximum viscosity and alters starch gelatinization and degradation, enhancing dough processing and fermentation. Mixolab results revealed significant variability in the rheological properties of flour–malt blends during heating and cooling, particularly in characterizing starch properties and enzymatic activity interaction. Significant differences were observed at C3, C4, and C5 stages, indicating that both malt concentration and diastatic power significantly influenced starch gelatinization (C3), liquefaction (C4), and retrogradation (C5). The decline in the gelatinization index (C3-C2) indicated a faster starch gelatinization process due to increased diastatic power, driven by heightened enzymatic activity. Higher diastatic powers showed a plateau effect, suggesting a saturation point in enzymatic activity. Increased malt concentration led to more rapid and extensive starch breakdown, closely related to the gelatinization and retrogradation processes in baking. The starch breakdown facilitated by the incorporation of malt increases maltose content, which is crucial for fermentation as it serves as a significant nutrient medium for microorganisms. The metabolic activity of these microorganisms is evidenced by a drop in pH and an increase in total acidity at the end of the fermentation process. A comprehensive understanding of malt’s enzymatic activity and its influence on flour properties allows for the establishment of frameworks to regulate and oversee the preparation processes for rye bread.

## Figures and Tables

**Table 1:** Rye flour characteristics.

Rye Flour Code	Falling Number (s)	Moisture (%)
RF1	346	13.79
RF2	262	14.65
RF3	210	14.25

**Table 2:** Diastatic rye malt characteristics.

Malt Code	Diastatic Power (°WK)	Moisture (%)
DP170	170	5.59
DP179	179	4.60
DP261	261	4.49
DP362	362	5.39
DP408	408	6.23

°WK—degrees Windisch–Kolbach.

**Table 3:** Ingredients used for scald preparation.

Ingredients	S-M0.5 (g)	S-M1.0 (g)	S-M1.5 (g)	Control (g)
Rye flour (RF2)	542	540	537	545
Water	800	800	800	800
Malt	3	5	8	–

RF2—rye flour (FN—262 s); Scald prepared with malt DP (170, 261, 408 °WK) in 0.5% concentration—S-M0.5, in 1.0%—S-M1.0, in 1.5%—S-M1.5; Control—sample without added malt.

**Table 4:** Reducing sugar content of malt and flour.

Reducing Sugar	RF2	DP170	DP261	DP408
	g 100 g^−1^	g 100 g^−1^	g 100 g^−1^	g 100 g^−1^
Fructose	0.08 ± 0.00	0.08 ± 0.00	0.07 ± 0.00	0.09 ± 0.00
Glucose	0.06 ± 0.02	0.75 ± 0.01	0.50 ± 0.00	0.67 ± 0.01
Sucrose	0.93 ± 0.03	2.31 ± 0.00	2.39 ± 0.08	3.18 ± 0.08
Maltose	0.05 ± 0.00	1.16 ± 0.03	0.82 ± 0.01	1.29 ± 0.05

RF2—rye flour (FN—262 s); DP170, DP261, DP408—malt with different DP (170, 261, 408 °WK).

## Data Availability

The original contributions presented in the study are included in the article/Supplementary Materials, further inquiries can be directed to the corresponding author.

## Supplementary Materials

The following supporting information can be downloaded at: https://www.mdpi.com/article/10.3390/foods13132077/s1, Table S1: Linear Models of Falling Number (FN, s) for Rye Flour–Malt Blends; Table S2: Mixolab values of rye flour and diastatic rye malt blends.
