# Supplementary material for: Impact of Rye Malt with Various Diastatic Activity on Wholegrain Rye Flour Rheology and Sugar Formation in Scalding and Fermentation Processes
Source: Foods. 2024 Jul 1;13(13):2077. doi: 10.3390/foods13132077 (PMC11241332; doi:10.3390/foods13132077)
Supplement: Supplementary file 1 [file foods-13-02077-s001.zip › foods-3077138-supplementary.pdf]

Table S1. Linear Models of Falling Number (FN, s) for Rye Flour-Malt Blends at Various Concentrations and Malt Activities for Samples RF1, RF2, and RF3.

**RF1**	**FN 346 (s)**	**Malt concentration (%)**										
	**FN (s)**	0.5	0.6	0.7	0.8	0.9	1.0	1.1	1.2	1.3	1.4	1.5
**Malt diastatic power (°WK)**	170	229	222	216	209	202	195	188	181	174	168	161
	190	224	218	211	204	197	190	183	176	169	163	156
	210	219	213	206	199	192	185	178	171	165	158	151
	230	215	208	201	194	187	180	173	166	160	153	146
	250	210	203	196	189	182	175	168	162	155	148	141
	270	205	198	191	184	177	170	163	157	150	143	136
	290	200	193	186	179	172	165	158	152	145	138	131
	310	195	188	181	174	167	160	154	147	140	133	126
	330	190	183	176	169	162	155	149	142	135	128	121
	350	185	178	171	164	157	151	144	137	130	123	116
	370	180	173	166	159	152	146	139	132	125	118	111
	390	175	168	161	154	148	141	134	127	120	113	106
	410	170	163	156	149	143	136	129	122	115	108	101
**RF2**	**FN 262 (s)**	**Malt concentration (%)**										
	**FN (s)**	0.5	0.6	0.7	0.8	0.9	1.0	1.1	1.2	1.3	1.4	1.5
**Malt diastatic power (°WK)**	170	194	189	184	179	174	169	164	159	154	149	144
	190	190	185	180	175	170	165	160	155	150	145	140
	210	187	182	177	172	167	162	157	152	147	142	137
	230	183	178	173	168	163	158	153	148	143	138	133
	250	179	174	169	164	159	154	149	144	139	134	129
	270	176	171	166	161	156	151	146	141	136	131	126
	290	172	167	162	157	152	147	142	137	132	127	122
	310	169	164	159	154	149	144	139	134	129	124	118
	330	165	160	155	150	145	140	135	130	125	120	115
	350	161	156	151	146	141	136	131	126	121	116	111
	370	158	153	148	143	138	133	128	123	118	113	108
	390	154	149	144	139	134	129	124	119	114	109	104
	410	150	145	140	135	130	125	120	115	110	105	100
**RF3**	**FN 210 (s)**	**Malt concentration (%)**										
	**FN (s)**	0.5	0.6	0.7	0.8	0.9	1.0	1.1	1.2	1.3	1.4	1.5
**Malt diastatic power (°WK)**	170	172	168	165	161	157	154	150	146	142	139	135
	190	170	166	162	158	155	151	147	144	140	136	132
	210	167	163	160	156	152	148	145	141	137	134	130
	230	164	161	157	153	150	146	142	138	135	131	127
	250	162	158	154	151	147	143	140	136	132	128	125
	270	159	156	152	148	144	141	137	133	130	126	122
	290	157	153	149	146	142	138	134	131	127	123	120
	310	154	150	147	143	139	135	132	128	124	121	117
	330	151	148	144	140	137	133	129	125	122	118	114
	350	149	145	141	138	134	130	127	123	119	115	112
	370	146	143	139	135	131	128	124	120	117	113	109
	390	144	140	136	133	129	125	121	118	114	110	107
	410	141	137	134	130	126	123	119	115	111	108	104

Table S2. Mixolab results of rye flour and diastatic rye malt blends. C1 (hydration), C2 (dough consistency), C3 (peak viscosity during heating – starch gelatinization), C4 (gel stability during cooling – amylase activity), and C5 (consistency of the dough during the cooling phase - starch retrogradation) (Nm). Gelatinization index (C3-C2), liquefaction index (C3-C4) and retrogradation index (C5-C4) (Nm).

**Malt DP (°WK)**	**0**	**170**	**170**	**170**	**179**	**179**	**179**	**261**	**261**	**261**	**362**	**362**	**362**	**408**	**408**	**408**
**Malt conc. (%)**	**0**	**0.5**	**1.0**	**1.5.**	**0.5**	**1.0**	**1.5.**	**0.5**	**1.0**	**1.5.**	**0.5**	**1.0**	**1.5.**	**0.5**	**1.0**	**1.5.**
**Rye flour 1**																
WA (%)	67.00	64.20	64.20	64.20	66.70	66.70	66.70	65.00	65.00	65.00	65.90	65.90	65.90	65.00	65.00	65.00
C1 (Nm)	1.13	1.11	1.10	1.08	1.09	1.08	1.08	1.10	1.09	1.09	1.11	1.15	1.06	1.13	1.13	1.13
C2 (Nm)	0.80	0.71	0.69	0.66	0.67	0.68	0.66	0.69	0.67	0.69	0.72	0.73	0.67	0.73	0.72	0.72
C3 (Nm)	2.28	2.14	2.03	1.96	2.01	1.90	1.81	2.03	1.92	1.80	2.04	1.93	1.77	1.99	1.86	1.77
C4 (Nm)	0.89	0.71	0.57	0.49	0.55	0.43	0.35	0.48	0.34	0.27	0.51	0.38	0.24	0.46	0.31	0.24
C5 (Nm)	1.45	1.21	1.00	0.84	1.03	0.78	0.61	0.86	0.63	0.45	0.93	0.67	0.42	0.82	0.55	0.41
C3-C2 (Nm)	1.53	1.43	1.34	1.30	1.35	1.22	1.15	1.34	1.24	1.12	1.32	1.20	1.10	1.26	1.14	1.05
C3-C4 (Nm)	1.39	1.43	1.47	1.47	1.47	1.47	1.47	1.55	1.58	1.53	1.54	1.55	1.53	1.53	1.55	1.52
C5-C4 (Nm)	0.55	0.51	0.43	0.35	0.48	0.35	0.26	0.38	0.29	0.18	0.42	0.29	0.18	0.37	0.24	0.17
**Rye flour 2**																
WA (%)	62.00	60.70	60.70	60.70	60.90	60.90	60.90	61.00	61.00	61.00	61.50	61.50	61.50	61.90	61.90	61.90
C1 (Nm)	1.14	1.13	1.12	1.08	1.13	1.10	1.14	1.14	1.10	1.13	1.13	1.14	1.14	1.12	1.12	1.07
C2 (Nm)	0.67	0.61	0.61	0.59	0.57	0.58	0.61	0.61	0.59	0.61	0.62	0.61	0.61	0.61	0.61	0.58
C3 (Nm)	2.45	2.30	2.13	1.97	2.06	2.00	1.91	2.06	1.83	1.79	2.04	1.88	1.76	1.99	1.82	1.66
C4 (Nm)	1.45	1.10	0.93	0.76	0.92	0.77	0.66	0.78	0.51	0.46	0.76	0.56	0.44	0.72	0.52	0.38
C5 (Nm)	2.05	1.71	1.50	1.24	1.45	1.20	1.07	1.28	0.86	0.76	1.26	0.95	0.72	1.24	0.86	0.62
C3-C2 (Nm)	1.79	1.69	1.53	1.38	1.49	1.42	1.30	1.45	1.24	1.18	1.42	1.27	1.15	1.38	1.21	1.08
C3-C4 (Nm)	1.01	1.20	1.20	1.21	1.13	1.23	1.25	1.28	1.32	1.33	1.28	1.32	1.32	1.27	1.30	1.28
C5-C4 (Nm)	0.60	0.61	0.57	0.48	0.53	0.43	0.41	0.50	0.35	0.30	0.50	0.40	0.27	0.52	0.34	0.24
**Rye flour 3**																
WA (%)	63.30	60.70	60.70	60.70	60.90	60.90	60.90	61.00	61.00	61.00	61.50	61.50	61.50	61.90	61.90	61.90
C1 (Nm)	1.13	1.13	1.12	1.11	1.09	1.10	1.13	1.11	1.12	1.15	1.11	1.10	1.10	1.07	1.13	1.12
C2 (Nm)	0.68	0.68	0.66	0.65	0.64	0.63	0.65	0.67	0.66	0.67	0.65	0.65	0.63	0.63	0.65	0.65
C3 (Nm)	2.15	2.15	2.10	2.03	2.08	1.98	1.93	2.08	1.96	1.89	2.05	1.91	1.83	1.99	1.87	1.76
C4 (Nm)	0.71	0.66	0.54	0.47	0.59	0.47	0.39	0.51	0.38	0.31	0.50	0.37	0.29	0.44	0.33	0.27
C5 (Nm)	1.21	1.03	0.86	0.76	0.94	0.76	0.63	0.80	0.60	0.49	0.81	0.57	0.46	0.69	0.54	0.42
C3-C2 (Nm)	1.47	1.47	1.44	1.38	1.44	1.35	1.28	1.41	1.30	1.23	1.40	1.25	1.20	1.36	1.22	1.11
C3-C4 (Nm)	1.43	1.50	1.56	1.55	1.49	1.51	1.54	1.57	1.58	1.58	1.55	1.54	1.53	1.55	1.54	1.49
C5-C4 (Nm)	0.50	0.37	0.33	0.29	0.35	0.29	0.24	0.29	0.22	0.18	0.31	0.21	0.16	0.26	0.20	0.15
